# Genetics and genomics of root system variation in adaptation to drought stress in cereal crops

**DOI:** 10.1093/jxb/eraa487

**Published:** 2020-10-23

**Authors:** Md Nurealam Siddiqui, Jens Léon, Ali A Naz, Agim Ballvora

**Affiliations:** 1 Institute of Crop Science and Resource Conservation (INRES) – Plant Breeding and Biotechnology, University of Bonn, Bonn, Germany; 2 Department of Biochemistry and Molecular Biology, Bangabandhu Sheikh Mujibur Rahman Agricultural University, Gazipur, Bangladesh; 3 University of Warwick, UK

**Keywords:** Cereals, comparative genomics, drought stress adaptation, genetic variations, molecular breeding, root system attributes

## Abstract

Cereals are important crops worldwide that help meet food demands and nutritional needs. In recent years, cereal production has been challenged globally by frequent droughts and hot spells. A plant’s root is the most relevant organ for the plant adaptation to stress conditions, playing pivotal roles in anchorage and the acquisition of soil-based resources. Thus, dissecting root system variations and trait selection for enhancing yield and sustainability under drought stress conditions should aid in future global food security. This review highlights the variations in root system attributes and their interplay with shoot architecture features to face water scarcity and maintain thus yield of major cereal crops. Further, we compile the root-related drought responsive quantitative trait loci/genes in cereal crops including their interspecies relationships using microsynteny to facilitate comparative genomic analyses. We then discuss the potential of an integrated strategy combining genomics and phenomics at genetic and epigenetic levels to explore natural genetic diversity as a basis for knowledge-based genome editing. Finally, we present an outline to establish innovative breeding leads for the rapid and optimized selection of root traits necessary to develop resilient crop varieties.

## Teaser

Exploiting root system variations related to drought stress adaptation using quantitative genetics and genomics approaches has potential in developing resilient varieties of cereal crops.

## Introduction

The adverse impacts of abiotic stresses are increasing owing to the rapid increase in climatic unpredictability and successive degradation of arable lands. This is negatively affecting the overall homeostasis of plants, and limiting crop expansion and, ultimately, crop production worldwide (Bray *et al.*, 2000; Checker *et al.*, 2012; Fahad *et al.*, 2017). Approximately 50–70% of the crop yield reduction is the direct consequence of abiotic stresses (Francini and Sebastiani, 2019). Drought is a major abiotic stress factor, significantly affecting crop yields by negatively affecting plant growth, physiology, and reproduction (Fahad *et al.*, 2017; Lamaoui *et al.*, 2018). For instance, a meta-study based on data published from 1980 to 2015 reported that up to 21% and 40% of yield losses on a global scale in wheat (*Triticum aestivum* L.) and maize (*Zea mays* L.), respectively, result from the negative effects of drought stress (Daryanto *et al.*, 2016).

Natural populations of crop plants, in terms of landraces and wild relatives, have established stunning levels of variation in developmental and adaptive traits. This has occurred through the continuous process of evolution and by-passing the bottlenecks of natural selection over an extremely protracted time span. These unique variations are essential sources of new traits that can be used to overcome yield stagnancy, improve climatic adaptation, and increase the narrow genetic diversity of cultivated varieties (Govindaraj *et al.*, 2015). Thus, the systematic genetic and molecular determination of natural genetic resources for crops across varied environments is essential. It will help to identify and incorporate new breeding targets for yield and sustainability that will help meet the current and future challenges of crop production and climate change. The power of quantitative genetics and genomics has increased considerably in the last decade after the advent of state-of-the-art molecular genome analyses methods, such as next-generation sequencing (NGS) and high-throughput genotyping (Mwadzingeni *et al.*, 2016, 2017). These methods have allowed the rapid identification of the hidden genetic footprints of complex traits, such as root system variation, with great precision (Shelden and Roessner, 2013; Naz *et al.*, 2014). In addition, phenomics is emerging as a new way to investigate plant traits at morphological and physiological levels under given environmental conditions using modern sensing and quantification techniques. Recently, newer methods, such as transcriptomics, proteomics, metabolomics, and ionomics (‘molecular phenotyping’), have been employed to determine trait inheritance at the physiological level. Similarly, non-invasive phenotyping methods are available to record system views of plant development, the energy dynamics of yield and yield components, and drought fitness from the cellular to whole-plant levels (Rascher *et al.*, 2011).

A plant’s root is the fundamental organ that plays critical roles in extracting soil resources under water-limited conditions. When plants sense a water shortage, roots continue growing and enter deep soil layers (Maeght *et al.*, 2013; Koevoets *et al.*, 2016; Fan *et al.*, 2017; Vyver and Peters, 2017). Root architecture and morphological attributes are crucial in the dehydration avoidance through efficient uptake of water and nutrients and favorable gas exchange, which facilitate carbon assimilation and yield potential under a drought stress scenario (Gewin, 2010; Kell, 2011; Lopes *et al.*, 2011; Palta *et al.*, 2011). Many studies have focused on the genetics of root system variations and its role in drought stress adaptation and yield stability (de Dorlodot *et al.*, 2007; Lynch, 2011; Uga *et al.*, 2015; Koevoets *et al.*, 2016; Polania *et al.*, 2017). The global genetic diversity may be readily exploited using marker-assisted selection (MAS) and genomic selection tools to produce elite cultivars able to face environmental extremes (Tron *et al.*, 2015; Valliyodan *et al.*, 2017). While numerous comprehensive reviews have highlighted the genetic diversity of root system architecture under drought conditions in legumes (Valliyodan *et al.*, 2017; Ye *et al.*, 2018), root crops, and tuber crops (Khan *et al.*, 2016), we focused on root system genomics and their utility in drought stress adaptation in cereal crops. In this review, we address the importance of harnessing the genetic variation in root system traits to promote adaptations to drought stress. It will be useful to address the following questions related to root system genetics and genomics involved in drought stress adaptation. (i) How do root system traits confer tolerance to drought stress by maintaining root–shoot balance? (ii) What is the current progress in genetic studies to identify genetic variations and genomic loci in response to drought stress? (iii) Which specific loci and syntenic regions are related to stress adaptation for resilience breeding? (iv) Which strategies will hasten the development of resilient cereal varieties through the employment of newly established techniques of phenomics, genomics, molecular breeding, and genome editing?

## Cereal productivity is threatened by drought stress worldwide

In recent years, drought stress has become the most vital cause of crop yield reductions (Lamaoui *et al.*, 2018; Webber *et al.*, 2018). Whole agroecosystems may suffer from frequent drought risks as the consequence of the global increase in temperature (Leng and Hall, 2019). According to the FAO (2018), >60% of the global population will inhabit areas with water deficiencies by 2025, and currently 70% of the world’s freshwater withdrawals are for agricultural purposes. With the dramatic increase in the global population, water requirements are rising at an alarming rate, resulting in an increasing need to breed water-efficient crops (Ruggiero *et al.*, 2017; Blankenagel *et al.*, 2018). The breeding of drought-tolerant cultivars requires improved root system architectural attributes as a necessity for the crops to cope with the changing climatic conditions.

Plants experience drought stress either when the water availability near the root zone is limited or when there is an imbalance between water uptake and loss through transpiration that hinders plant growth and development during the plant life cycle (Ji *et al.*, 2010; Anjum *et al.*, 2011). Under drought stress conditions, plants exhibit a wide variety of disorganization that may result in an alteration from high sensitivity to viable tolerance (Joshi *et al.*, 2016). Drought restricts various crucial physiological processes, including growth performance, correlations between nutrients and water, photosynthesis, and assimilate partitioning, which consequently results in signiﬁcant reductions in biomass production and yield (Daryanto *et al.*, 2017; Hussain *et al.*, 2018). A lower absorption rate of photosynthetically active radiation, a decreased radiation use efficiency, and a decline in the harvest index are leading factors of yield reduction under limited soil moisture conditions (Earl and Davis, 2003; Randhawa *et al.*, 2017). Evolution has shaped the inherent potential of plant populations for morphological and physiological adjustments that mitigate the detrimental impacts of drought stress (Farooq *et al.*, 2009; Basu *et al.*, 2016).

Drought tolerance has been defined as the ability of certain genotypes to perform better than others under drought stress conditions. The underlying mechanisms may involve dehydration tolerance or avoidance, as well as drought escape (Levitt, 1972; Turner, 1979; Blum, 2005). Therefore, the extent and combination of these adaptive mechanisms need to be explored to understand the utility levels of different traits in enhancing the tolerance to water scarcity of crop varieties. Plant physiologists have explored several emerging root–shoot system traits that might trigger plant adaptation to drought stress by improving the water use efficiency, as well as a limited number of traits that may help optimize soil water acquisition (Choat *et al.*, 2018; Rosa *et al.*, 2019).

## Interplay of root and shoot attributes in increasing cereal yield and sustainability under drought stress conditions

**Fig. 1.**
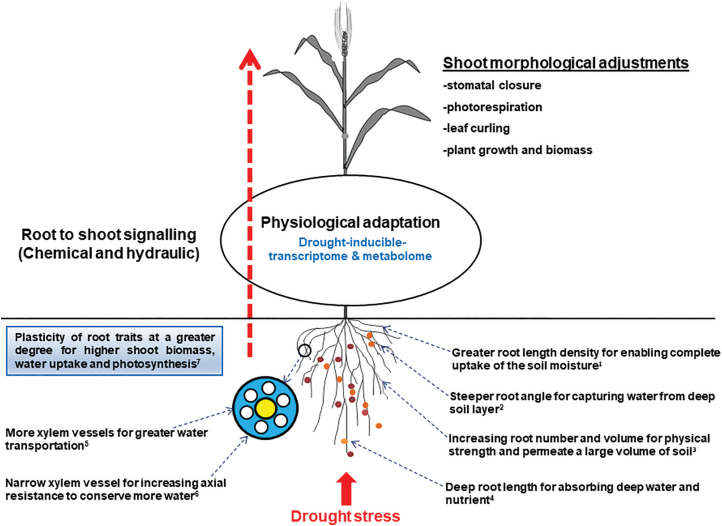
Diagram of plant root–shoot system revealed functions of root system attributes to improve shoot morphological adjustment through root–shoot signaling under drought stress conditions (modified from Reinert *et al.*, 2019). (^1^Lynch, 2013; ^2^Uga *et al.*, 2011; ^3^Sharma *et al.*, 2011; ^4^Wasson *et al.*, 2014; ^5^Wasson *et al.*, 2012; ^6^Richards and Passioura, 1989; ^7^Kano-Nakata *et al.*, 2011).

Roots and shoots are two fundamental axes of plant development. Although roots and shoots develop and grow at different locations, there is an active communication between the two organs that determines the specific plant architecture (Fig. 1). Interconnected hormonal circuits, as chemical signaling pathways, dominated by auxin and cytokinins play fundamental roles in the coordinated development of these organs (Puig *et al.*, 2012; Naz *et al.*, 2013). There exists a rootwards auxin flow from the shoot and a shootwards cytokinin flow from the root (Ko *et al.*, 2014). The auxin transported from shoot to root activates strigolactones in the roots that then move upwards through the xylem and suppress axillary shoot branching (tillering). Additionally, shoot biomass is heavily influenced by above-ground environmental factors (such as photoperiod, rainfall, and temperature). Similarly, roots show a great plasticity in responses to available soil moisture and nutrients, as well as in their interactions with biota in the rhizosphere (Zhu *et al.*, 2011; Chen *et al.*, 2019). The root, shoot, and atmospheric factors combine to influence plant adapative systematic responses, including stomatal closure, to diverse environmental stimuli, including drought stress (Jia and Zhang, 2008). Interestingly, shoot growth is reduced under drought stress conditions but root growth continues through essential reserve translocation from the shoot using long-distance chemical and hydraulic signal transduction (Fig. 1; Davies *et al.*, 2002; Schachtman and Goodger, 2008).

Unlike the tap root system in dicots, major cereal crops establish a fibrous root system that comprises two components, seminal and nodal roots (Lucas *et al.*, 2000; Manske and Vlek, 2002; Smith and Smet, 2012). Seminal roots develop during post-embryogenesis from embryo radicals, while nodal roots are initiated at the base of each established tiller during ontogeny (Wahbi, 1995). The development of each tiller above ground consequently increases the number of nodal roots below ground because of their location close to the soil. The development of nodal roots in turn enhances the uptake of water and nutrients, as well as favorable gas exchanges, which facilitate the initiation of new tillers and shoot growth (Naz *et al.*, 2014). A direct positive correlation has been reported between root and shoot traits in barley (Arifuzzaman *et al.*, 2014; Naz *et al.*, 2014). However, it is still largely unknown whether shoots facilitate the production of more nodal roots or the increase in rooting influences tillering and additional shoot attributes positively. The answer to this question is of fundamental importance in understanding the interplay between root and shoot attributes to establish desirable plant architectures of cereal crops and to use potentially positive genetic and environmental interactions to increase yield and sustainability.

## How do root system traits mediate tolerance to drought stress?

Roots form indispensable biological plant structures that largely contribute to the plant’s ability to recover from drought stress. A ‘deep, wide-spreading, much-branched root system’ is a crucial landmark of drought tolerance as stated by Kramer (1969). The depth and spreading nature of root systems are recognized as the key components that allow plants to access available soil water (Blum, 2011; Fenta *et al.*, 2014), and their advantageous effects on adaption to drought stress have been determined in many economically important cereal crops.

Root angle is considered an important drought-adaptive trait that determines the horizontal and vertical distributions of roots into the soil (Christopher *et al.*, 2013; Uga *et al.*, 2013a). Root angle has been intimately linked with the deep rooting reported in rice (Kato *et al.*, 2006), wheat (Slack *et al.*, 2018, Preprint), and sorghum (Singh *et al.*, 2011). Narrower root angles may decrease the energy supplied during root penetration into the deeper soil horizons to optimize water uptake under limited rainfall conditions (Fig. 1; Wasson *et al.*, 2012; Meister *et al.*, 2014; Oyiga *et al.*, 2019). Deep rooting in thinner root systems, compared with thick or shallow root systems, has the potential to adjust to the soil components, particularly in the water-limited dry land soils (Lynch, 2014). Therefore, cultivars possessing greater primary root elongation, a lower lateral root branching tendency, and extensive root hairs are more likely to access soil moisture from deep soil layers under water shortage conditions (Wasson *et al.*, 2012; Uga *et al.*, 2013a; Akman and Topal, 2014; Lynch *et al.*, 2014). Root architectural traits are also characterized by proliferative roots developed through lateral root initiation and elongation, and these characteristics include lateral root number/volume, root length density, and root surface area, which aid in water uptake from water-limited soils (Fig. 1; Ye *et al.*, 2018). Greater root masses and root length densities improve yield performances by enhancing the water uptake rate when the subsoil layers have limited water, but they are negatively associated with grain yield when present in topsoil layers (Fang *et al.*, 2017). Another important component of a proliferative root system is root surface area, which represents the total area of the root system that is in contact with the soil, and an increase in area improves drought stress tolerance (Sharma *et al.*, 2011; Wasson *et al.*, 2012).

In addition to root architectural traits, a wide range of root anatomical traits, such as cell size, number, configuration, and density, determine the pathways through which water and nutrients enter and are transported (Marschner, 1995; Burton *et al.*, 2013). Other crucial anatomical traits, such as cell wall thickness and cell density, provide mechanical strength to the root system during severe environmental stresses (Justin and Armstrong, 1987; Striker *et al.*, 2007). A modification of the root anatomy, such as aerenchymal development in maize (Lynch, 2011; Burton *et al.*, 2013), may store the energy supply to accelerate soil exploration and penetration during water stress conditions (Addington *et al.*, 2006; Maseda and Fernández, 2006). The size of the root xylem vessels is correlated with drought tolerance in cereals, and a reduction in the diameters of xylem vessels increases the amount of water extracted per unit of root length (Fig. 1; Giuliani *et al.*, 2005; Comas *et al.*, 2013). The diameters and distribution of the xylem vessels, especially metaxylem that regulates root axial hydraulic conductivity, have been reported to affect drought stress tolerance in cereal crops (Kadam *et al.*, 2015, 2017). Importantly, useful anatomical traits, such as root cortical aerenchymae, cortical cell size, and the cortical cell file number, help limit the nutrient and carbon costs of soil exploration by altering root cortical tissues to air spaces (Lynch, 2013).

To cope with drought stress, cereal species tend to be plastic (Kadam *et al.*, 2017). The phenotypic plasticity of a genotype against rapid climatic fluctuations and severe drought stress requires an integrated response by different drought stress-adaptive mechanisms, such as dehydration resistance and dehydration escape or avoidance (Levit, 1972; Kadam *et al.*, 2017). Recently, several studies have shown that plasticity of root traits is mostly advantageous for the effective adaptation to drought stress (Kadam *et al.*, 2015; Sandhu *et al.*, 2016; Schneider *et al.*, 2020a, b). For instance, under drought stress conditions, the plasticity of different root traits, such as root length density and total root length (Kano *et al.*, 2011; Kano-Nakata *et al.*, 2011, 2013; Tran *et al.*, 2015), contributed to a greater shoot biomass and increased water use and photosynthetic efficiency levels (Fig. 1). The plasticity of root system responses also induces tolerance to drought stress by increasing the number of fibrous roots, and minimizing the lateral root diameters and root biomass fluctuations (Osmont *et al.*, 2007; Meister *et al.*, 2014; Salazar-Henao *et al.*, 2016). Recently, the genomic loci regulating root phenotypic plasticity under drought stress conditions in cereal crop species (Sandhu *et al.*, 2016; Kadam *et al.*, 2017; Schneider *et al.*, 2020a, b) revealed that the plasticity of root systems might be an excellent source of genetic variation for stress adaptation (Schneider and Lynch, 2020).

## Genetics of root system variation and drought stress adaptation in cereal crops

Modifications of the root system resulting from natural domestication and breeding have led to differing root architectural spatial configurations (de Dorlodot *et al.*, 2007). Therefore, the identification of novel quantitative trait loci (QTLs) is a fundamental research platform in the dissecting of the large genetic variabilities of root system attributes. Genome-wide mapping has been employed to identify novel genetic loci for root architectural traits using different mapping populations, such as introgression and recombinant inbred lines (RILs), biparental populations, and global core collections. In the following sections, we summarize the identification of essential QTLs/genes for root-related drought stress adaptation in selected cereal species.

### Rice

Rice is widely cultivated on lowland rainfed and irrigated areas in Asia and Africa as an essential component of subsistence farming. Rice is highly susceptible to drought stress, and even moderate drought stress may cause significant yield losses in rice (O’Toole, 1982; Centritto *et al.*, 2009). In cereals in general, but especially in rice, deep rooting is determined by the distribution of wide root growth angles and root lengths (Uga *et al.*, 2015). An enormous genetic variation in root angle distribution has been identified in two root system categories; group A has shallow rooting and group B has shallow to deep rooting on the basis of the characterization of 97 rice accessions (Tomita *et al.*, 2017). Six major effect QTLs for deep rooting were unraveled (Uga *et al.*, 2011, 2012, 2013*b*, 2015; Kitomi *et al.*, 2018) using RILs derived from a cross between ‘IR64’, a lowland cultivar possessing a shallow rooting system with an inactive *DEEPER ROOTING 1* (*DRO1*) allele, and ‘Kinandang Patnog’, an upland cultivar possessing a deep rooting system with an active *DRO1* allele, implying the involvement of the locus in the deep root phenotype (Uga *et al.*, 2011; Kitomi *et al.*, 2018). Similarly, another study identified a key QTL for root growth angle (*DRO2*) on chromosome 4 using three F_2_ populations derived from crosses between each of three shallow rooting cultivars (‘ARC5955’, ‘Pinulupot1’, and ‘Tupa729’) and ‘Kinandang Patong’ (Uga *et al.*, 2013*b*). On the basis of their findings, we concluded that ‘Kinandang Patong’ has contributory *DRO1* and *DRO2* alleles that direct downward rice root growth. Kitomi *et al.* (2018) also identified two major QTLs for root length, *QUICK ROOTING 1* (*QRO1*) on chromosome 2 and *QRO2* on chromosome 6, using the same parents. High-throughput phenotyping and genotyping of a rice-mapping population of 361 diverse lines derived from a cross between ‘Moroberekan’ and ‘Swarna’ were carried out to map QTLs for drought tolerance traits, including root architecture. The identified drought yield QTL, *qDTY3.2*, for deeper root growth contributes to sustaining the entire plant’s water status by interacting with shoot-related drought tolerance traits (Grondin *et al.*, 2018).

**Fig. 2.**
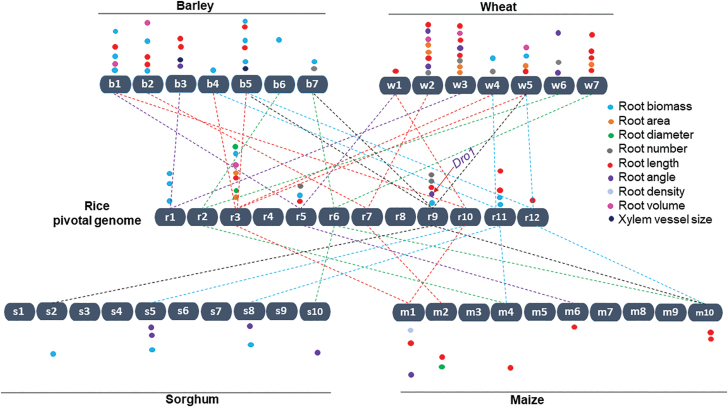
Schematic representation of the cereal genome-wide microsynteny map showing the effect of major QTLs for root system attributes identified between rice chromosomes (r1–r12) adopted as a reference, and the wheat (w1–w7), barley (b1–b7), maize (m1–m10), and sorghum (s1–s10) genomes in response to drought stress. The microsynteny map was constructed based on Salse *et al.* (2009). Each line represents an orthologous locus for root-related drought-adaptive traits highlighted across the genomes. The approximate chromosomal locations of the QTLs are represented by different colors based on published reports. Detailed information and accurate positions of the QTLs are provided in Supplementary Table S1.

The genetic mapping of rice germplasms in response to drought stress has identified genomic loci associated with root traits (Fig. 2; see Supplementary Table S1 at *JXB* online). A meta-study resulted in the identification of a valuable genomic region, the ‘QTL hotspot’, harboring meta-QTLs associated with root architectural traits, such as root density, maximum root elongation and thickness, root/shoot ratio, and the root penetration index, as well as drought tolerance traits. The ‘QTL hotspot’ is a demarcated segment of 5 Mb that encompasses only a few stress-related candidate genes (Courtois *et al.*, 2009). Another study which investigated root traits of the seedlings of 162 F_12_ RILs derived from a cross between ‘Milyang23’ and ‘Tong88-7’ identified five major QTLs related to important root traits, such as root length, root dry weight, and dry weight (Fig. 2; Han *et al.*, 2018). The alleles for the QTLs donated by ‘Tong88-7’ contributed to the improvement in the root traits. The QTLs involved in drought tolerance and root architecture-related traits have been mapped using composite interval mapping of a cross of ‘IR55419-04’ and ‘Super Basmati’. Three QTLs on chromosomes 3, 9, and 11 for deep rooting length, and four QTLs on chromosome 3 for deep root surface area and diameter, were identified under water deficit conditions, explaining 3.8–32.09% of the genetic variance. These three QTLs (*qDRL3*, *-9*, and *-11)* for deep rooting length have donor alleles from ‘IR55419-04’ (Fig. 2; Sabar *et al.*, 2019). Moreover, to harness the genetic variations in root architectural traits under drought conditions, an in-depth genome-wide association study (GWAS) identified 106 significant loci from a diverse panel of 274 *indica* genotypes. This included *a priori* candidate genes for regulating the water deficit stress plasticity of root architectural and anatomical characteristics (Kadam *et al.*, 2017). More recently, another study identified 143 loci contributing to 21 root traits, such as maximum root length, root volume, and root dry weight, under drought conditions. In total, root-related candidate genes, including *DRO1*, *WOX11*, and *OsPID* co-located with the associated loci, were identified (Li *et al.*, 2017).

### Wheat

As the second most economically important grain crop, wheat has been investigated in depth for root system variation. Recently, several advanced spring wheat accessions screened from the Cultivated Wheat Collection germplasms showed efficient root systems for extensive deep rooting and large root biomasses (Narayanan *et al.*, 2014). The geographic area from which wheat genotypes were collected had significant influences on rooting depth. The wheat subsets originating from Australia, the Mediterranean, and western Asia have greater rooting depths in comparison with subsets collected from south Asia, Latin America, Mexico, and Canada. The increased rooting length might be attributed to the adaptive traits of genotypes cultivated in the relatively drier environments of the western USA to enhance water acquisition. In a biparental RIL population derived from a cross between Chinese winter wheat varieties ‘Xiaoyan 54’ and ‘Jing 411’, two major QTLs were identified for primary root length and maximum root length, with the donor alleles being contributed by the older cultivar ‘Xiaoyan 54’, which has a larger and deeper root system (Ren *et al.*, 2012). A study by Ma *et al.* (2017) detected 15 QTLs on eight chromosomes related to root traits, including maximum root length and total root length, in a population of ‘Q1028’ and ‘ZM9023’. The positive QTL alleles were contributed from the semi-wild parent ‘Q1028’, which possesses a longer root system.

Genetic studies to explore the major QTL effects on root architectural traits under different water regimes have been carried out in wheat mapping populations. Liu *et al.* (2013) mapped seven consistently expressed QTLs that were associated with seminal root traits, including total root length, seminal root number, project root area, root surface area, and seminal root angle, and the individual QTLs manifested phenotypic variations ranging from 4.98% to 24.31% under different water regimes. This study importantly noticed that one chromosomal region at the interval *Xgwm644.2–P6901.2* on chromosome 3B harbored nine QTLs affecting most of the root morphological traits. Recently, favorable alleles of eight QTLs linked to root length were mapped to the wheat RILs derived from a cross between ‘W7984’ (synthetic) and ‘Opata 85’ under hydroponic conditions, with two of the eight QTLs being contributed from the drought-resistant parent ‘W7984’ (Fig. 2; Supplementary Table S1; Ayalew *et al.*, 2017). A GWAS of 91 phenotypically diverse genotypes across 21 countries displayed two significant drought induced major alleles that cause long root lengths under polyethylene glycol (PEG)-induced water stress. The GWAS approach also identified three drought-responsive pleiotropic single nucleotide polymorphism (SNP) markers associated with root dry biomass in a panel of 100 bread wheat genotypes selected on the basis of their breeding history for drought tolerance (Mathew *et al.*, 2019). Another study identified five significant markers causing extended rooting lengths under drought stress conditions using a mapping population consisting of two introgressed populations (Bhatta *et al.*, 2018).

### Barley

Drought stress is a detrimental limiting factor for barley that causes up to 50% yield reductions (Jenks and Hasegawa, 2005; Samarah, 2005). Roots of 301 ‘BC2DH’ populations derived from a cross between exotic accessions of *Hordeum vulgare* ssp. *spontaneum* C. Koch (ISR42-8) from Israel and the spring barley cultivar ‘Scarlett’ (*H. vulgare* ssp. *vulgare*) from Germany were sampled from the drought-induced tunnels at the mature stage and analyzed for root and other important physiological traits, including yield. When investigating favorable drought-responsive QTLs, Sayed (2011) found that the wild parent donated the favorable alleles to 27 (34.1%) of the 79 QTLs that influenced root and physiological traits. These novel exotic alleles contribute to drought-adaptive traits, such as root length and proline content. For instance, the presence of exotic alleles at marker locus *VrnH1* resulted in an extension of the root length by 9.17% under drought stress conditions. This result implied that the introgression from wild barley promoted longer root lengths in the ‘S42’ population. Using these introgression lines, other studies identified seven QTLs associated with root architectural traits, with the introgression of exotic alleles at the *QRl.S42.5H* loci accounting for a 9% increase in root length (Fig. 2; Supplementary Table S1; Arifuzzaman *et al.*, 2014). Naz *et al.* (2014) reported six major QTLs for root length, eight for root dry weight, and five for root volume, and all the beneficial QTL alleles of wild origin have been fixed in the ‘Scarlett’ cultivar background (Fig. 2). Moreover, high-throughput GWAS mapping for detecting QTLs associated with root architectural traits has been reported in barley recently. An association mapping study by Reinert *et al.* (2016) using 179 diverse genotypes, comprising 48 wild accessions and 131 cultivars, across 38 countries identified two drought-adaptive QTLs for root dry biomass on chromosomes 2H and 5H. By comparing their relative performances, a potential QTL (*QRdw.2H*) was identified on chromosome 5H at 95 cM, where the homozygous major allele produced the greatest variability on the phenotype (*R*^2^=24.93%) (Fig. 2; Supplementary Table S1; Reinert *et al.*, 2016). A panel of 233 barley genotypes containing a majority of the lines (223) from a worldwide broad genetic and phenotypic diversity panel, in which 58% of the genotypes were two rowed and 42% were six rowed, were analyzed for root architecture traits under drought stress conditions using a recently developed high-throughput phenotyping method. This study precisely identified a catalog of QTL-harboring ‘hotspots’ and four QTLs for drought-inducing root traits (Fig. 2; Supplementary Table S1; Abdel-Ghani *et al.*, 2019). Another recent study performed a comprehensive GWAS across three cropping seasons using a 192 diverse spring barley panel to characterize both root morphological and anatomical traits under water deficit stress. Three to four QTL intervals showed strong effects across growing seasons for both root morphological and anatomical traits in response to water deficit stress (Fig. 2; Supplementary Table S1; Oyiga *et al.*, 2019).

### Maize

Maize originated in a semi-arid area, where it grew in less fertile soil that lacked sufficient irrigation and fertilizers. Thus, improving the stress resilience of maize is necessary for continued crop production in these less cultivable areas. Large genetic diversity and heritability levels were found for maize root system traits (Burton *et al.*, 2014), ranging from small and compact to large and exploratory patterns, in a RIL nested association mapping (NAM) subpopulation derived from a cross between ‘B73’ (compact root system) and ‘Ki3’ (exploratory root system) (Zurek *et al.*, 2015). Clusters of QTLs for both root depth and average root width were mapped on chromosomes 2, 9, and 10, with the large additive effects on root depth and average root width originating from the ‘Ki3’ allele. QTL mapping using 187 ‘BC_4_F_3_’ maize lines derived from an interspecific cross between a larger root system donor parental line (‘Ye478’) and a small root system recurrent parental line (‘Wu312’) revealed 30 QTLs for root architectural traits, with 80.6% carrying a favorable allele originating from the donor parent ‘Ye478’ (Cai *et al.*, 2012). A single QTL was detected for drought-related traits using two inbred parents from drought-tolerant and -sensitive populations, and root density contributed 24% of the phenotypic variation (Fig. 2; Supplementary Table S1; Rahman *et al.*, 2011). Recently, a study identified major QTL effects for crown root angle (*CRA2*) and crown root length (*CRL1*) under drought conditions using a RIL population comprising 204 F_8_ lines derived from a cross between two inbred lines, ‘DH1M’ and ‘T877’ (Fig. 2; Supplementary Table S1; Li *et al.*, 2018). The ‘T877’ allele contributed a major effect on root angle at an SNP marker (288.8 cM), whereas the favorable allele for primary root length was from ‘DH1M’. Additionally, an in-depth GWAS was performed to identify SNPs for the most important root functional and structural traits, including rooting depth, root length, and root length density, related to drought-adaptive mechanisms using the CIMMYT Asia association mapping panel, consisting of 396 diverse inbred maize lines derived from tropical and subtropical pools and populations from the Latin American, African, and Asian maize programs. The CIMMYT lines were drought tolerant. In total, 18 SNPs were identified from manually and digitally scored root functional and structural traits that showed common associations with more than one trait. Of these, 12 SNPs were observed within or near the various gene functional regions (Zaidi *et al.*, 2016). A few recent field-based GWAS approaches pinpointed candidate genes for drought stress and environmental plasticity of root architectural and anatomical phenes using a large association panel comprised of maize inbred lines. The report showed that root phenotypic plasticity was highly quantitative, and plasticity loci were distinct from the loci that govern trait expression under water deficit and environmental stress conditions (Schneider *et al.*, 2020a, b).

### Sorghum

Sorghum is widely cultivated in tropical and subtropical semi-arid regions, mostly under natural soil moisture conditions. Large genetic diversity levels in root and shoot traits associated with drought stress were observed in 141 F_6_ RILs from a cross between two parents possessing a narrow and a wide angle for the first flush of nodal roots (Mace *et al.*, 2012). In this study, nodal root angle was significantly correlated with shoot traits, and four major QTLs for nodal root angle (*qRA*) were also successfully identified, which together explained 58.2% of the phenotypic variation (Fig. 2; Supplementary Table S1).

## Comparative genomics of root-related drought stress adaptation using microsynteny among cereal crop species

Evolving from a common ancestor, cereal crops revealed a significant genetic conservation among themselves (Salse *et al.*, 2008). This genetic conservation can be traced among the species using molecular (genomic) data and full-length genome sequence data for cereal crops. This concept also led to the establishment of inter-specific hybrid genome maps to identify syntenic chromosomal regions precisely across genomes as well as their interspecies variation. Here, we showed a comparative genomic map of cereal crops that revealed their genetic synteny of genome-wide QTLs/loci for root attributes related to drought stress adaptation (Fig. 2).

To date, 23, 31, 24, 9, and 7 major QTL effects on the different root traits in response to drought stress have been identified across the chromosomes of rice, wheat, barley, maize, and sorghum, respectively (Fig. 2; Supplementary Table S1). Interestingly, rice chromosomes r6, r9, and r11 were found to be syntenic with wheat chromosomes w7, w5, and w4, respectively, and barley chromosomes b7, b5, and b4, respectively, and they also had syntenic relationships with maize chromosomes m10, m10, and m4, respectively, and sorghum chromosomes s10, s2, and s8, respectively (Fig. 2). These syntenic regions revealed drought-adaptive QTLs for various root system attributes (Fig. 2), predicting a conserved genetic regulation among cereal genomes. Such synteny may facilitate an understanding of genome-wide relationships among QTLs/genes related to stress adaptation. More importantly, the unique cloned and characterized drought-adaptive rice gene *DRO1*, which regulates root growth angle, showed a high-yield performance under drought stress conditions (Uga *et al.*, 2013a). This gene lies on rice chromosome r9, which showed a syntenic relationship with wheat chromosome w5, barley chromosomes b5 and b7, maize chromosome m10, and sorghum chromosome s2, and all the syntenic chromosomal regions revealed associations with root-related drought stress adaptation (Fig. 2). This genome-wide syntenic relationship implies that other economically important cereal crops, such as wheat, barley, maize, and sorghum, contain *DRO1* homologs that might be useful for promoting root-related drought stress adaptations in cereals using comparative genomics. Furthermore, it will aid in the utilization of the genetic potential of crop species for particular adaptive responses to alter a specific mechanism in another species using natural genetic variations. However, the number of commonalities does not correspond to the genetic conservation. Such gaps may result from limited studies in one or the other cereal species because of large root phenotyping. Therefore, based on this microsynteny map, new studies should focus on root system characterization and genomics that may provide distinct genetic loci or genetic mechanisms among cereal crop species.

## Directions of future research on root system variations in drought stress adaptation and their introduction into breeding programs

Roots and shoots evolved together for nearly 3.5 million years. However, owing to directional selection for yield in the past century, root attributes were completely neglected in breeding programs, unless the improvement was indirect. Therefore, a future breeding dimension should focus simultaneously on the recruitment of lost root system variations for yield and sustainability.

Several studies reported that root system attributes enhance shoot architecture for yield and drought fitness in cereals, reflecting that roots should be the foremost breeding target of the future (Naz *et al.*, 2014; Sandhu *et al.*, 2016; Li *et al.*, 2018). The natural genetic diversity in differential root system architecture may be useful to understand drought adaptation mechanisms and improve cultivars by generating beneficial root architecture. To date, studies have reported and validated QTLs associated with root system traits, such as root length, biomass, number, angle, volume, diameter, density, and xylem vessel size, under drought stress conditions (Fig. 2; Supplementary Table S1). More importantly, the diversity of the wild relatives of crops showed remarkable root system variations that have great potential in drought stress adaptation (Naz *et al.*, 2014; Reinert *et al.*, 2016). Here, we summarized a considerable amount of donor genotypes, including wild relatives (Supplementary Table S1), but very limited strategies have been undertaken to exploit these lines in resilient breeding programs. These promising donor parents need to be introgressed into elite backgrounds to enhance the stress-adaptive potential of the cultivated gene pool.

The enhancement of root-related drought stress adaptation by applying classical breeding is difficult owing to the complexity levels of these traits (Witcombe *et al.*, 2007; Van Oosten *et al.*, 2016). Genomic and phenomic approaches are gaining popularly as important tools that allow in-depth analyses of crops to increase our understanding of the complexity of the mechanisms underlying stress adaptation. Although both *cis*- and *trans-*genetic components, along with epigenetics, are involved directly in trait complexity, the role of *cis*-genetic modules appears to be more influential on the quantitative divergence in expression of genes controlling polygenic traits across dynamic environments (Signor and Nuzhdin, 2018). Therefore, genotype×environment interactions form the biggest challenge in the precise genetic determination of these traits under field conditions. This scenario demands an expression QTL analysis as a high-resolution genomics approach for the precise dissection of traits at morphological and physiological levels across varying environments. In addition, over the last decades, NGS and bioinformatics tools have been rapidly advancing, allowing the discovery of new genes and regulatory sequences controlling diverse complex traits (Taunk *et al.*, 2019).

Comparative genomics is another reliable cutting-edge avenue that has increased the amount of available genomic information. Comparative genomic analyses characterize genomic structural alterations, gene structures, and genome synteny, as well as induced functions among cereal crop species. Using comparative genomics tools, stress-responsive differentially expressed genes regulating root system architectural traits have been identified in rice (Lou *et al.*, 2017), wheat (Dalal *et al.*, 2018; Hu *et al.*, 2018), maize (Li *et al.*, 2017), and barley (Kwasniewski *et al.*, 2016). For example, transcriptome profiling in rice identified 49 candidate differentially expressed genes, and a weighted gene co-expression network analysis confirmed 18 hub genes, all of which were more highly expressed in deep roots than in shallow roots (Lou *et al.*, 2017). Genetic modifications represent another viable option for crop advancement (Hussain, 2015; Mwadzingeni *et al.*, 2017). They provide the unique opportunity to edit the targeted genome sequences for particular breeding aims. Genome editing was recently revolutionized by CRISPR/Cas9 [clustered regularly interspaced palindromic repeats (CRISPR)/CRISPR-associated protein 9]-based approaches. As an advanced breeding tool, the CRISPR/Cas9 system has been successfully utilized to develop novel variants of *ARGOS8* by editing its promoter sequences to increase expression, which enhanced maize yield potentials under natural field drought stress conditions (Shi *et al.*, 2017). The above high-resolution genetics and genomic approaches may help dissect trait complexity and target selection in breeding (such as genomic selection), as well as functional analyses of genes controlling root-borne shoot dynamics and crosstalk in the determination of yield potentials in cereals.

In summary, we propose to implement the following multistep root breeding strategies to establish drought stress adaptations in cereals: (i) screening global genetic diversity levels using diverse natural populations to identify morphological and physiological novelties for root–shoot attributes related to yield and sustainability; (ii) establishing state-of-the-art populations for the high-resolution and quantitative dissection of traits; (iii) using high-throughput non-invasive and automated tools for root phenotyping under field conditions; (iv) combining high-resolution phenotypic trait data with genome-wide molecular data to identify QTL epistasis and gene×environment interactions using state-of-the-art computing models; (v) analyzing sequences of wild, landraces, and donor lines to dissect allelic variations associated with root-related drought stress adaptations using comparative genomics; (vi) deploying stably expressed major QTL effects and ‘QTL hotspot’ regions, as well as genomic selection tools, that integrate phenotype, genotype, and environment to improve breeding stocks; (vii) forwarding large effect QTLs both individually and by pyramiding QTLs for the functional characterization of the underlying genes; (viii) characterizing genetic synteny across the cereal genomes and developing interspecies hybrid genetic maps for gene isolation, comparative analyses, and interspecies introgression; (ix) employing expression QTL analyses based on RNA-sequencing as molecular phenotypes for high-resolution gene trait analyses; (x) manipulating and editing gene functions using technologies such as CRISPR/Cas9; (xi) establishing a link between quantitative traits and epigenetic signatures to reveal major roles in drought stress adaptation; and (xii) establishing an interdisciplinary research platform among geneticists, breeders, biotechnologists, agronomists, and crop physiologists to combine knowledge on root system variation. These efforts will allow us to focus on the most relevant traits, their combinations, and interplay in breeding programs to develop resilient crops and to secure sustainable cereal production under changing climatic conditions.

## Supplementary data

The following supplementary data are available at *JXB* online.

Table S1. List of recently identified major effect QTLs/genes associated with root system attributes to drought stress in major cereal crops.

eraa487_suppl_Supplementary_Material
