# Supplementary material for: Genetics and genomics of root system variation in adaptation to drought stress in cereal crops
Source: J Exp Bot. 2020 Oct 23;72(4):1007–19. doi: 10.1093/jxb/eraa487 (PMC7904151; doi:10.1093/jxb/eraa487)
Supplement: eraa487_suppl_Supplementary_Material [file eraa487_suppl_supplementary_material.pdf]

Supplementary Information

Title: Genetics and genomics of root system variation in adaptation to drought-stress in cereal crops

Author list

Md. Nurealam Siddiqui, Jens Léon, Ali A. Naz and Agim Ballvora

This PDF include:

Supplementary Table S1. List of recently identified major effect QTL/genes associated with root system attributes to drought stress in major cereal crops.

Table S1. List of recently identified major effect QTL/genes associated with root system attributes to drought-stress in major cereal crops.

Cereals	Mapping populations	Trait category	Root trait	QTL/genes	Donor parent	Chr. No.	Position (cM)	PVE	Reference
Rice	Milyang23×Tong88-7	Root length	Root length	*qRL11*	Tong88-7	11	16.0	8.97	Han *et al.,* 2018
		Root biomass	Root fresh weight	*qRFW9*	Tong88-7	9	14.0	10.06	Han *et al.,* 2018
				qRFW11	Tong88-7	11	9.0	4.32	Han *et al.,* 2018
			Root dry weight	*qRDW11*	Tong88-7	11	13.0	8.35	Han *et al.,* 2018
	IR64-21×Dular	Root number	% of deep root	*qRT9.1*	Dular	9	53.2	-	Catolos *et al.,* 2017
			% of shallow root	*qRT9.1*	IR64-21	9	53.2	-	Catolos *et al.,* 2017
			Crown root number	*qRT5.1*	-	5	63.9	-	Catolos *et al.,* 2017
		Root length	Root length	*qRT5.1*	-	5	58.9	-	Catolos *et al.,* 2017
		Root biomass	Root dry weight	*qRT5.1*	-	5	59.9	-	Catolos *et al.,* 2017
	Cocodrie×N-22	Root length	Root length	*qRL12.04*	N-22	12	30.0	5.1	Bhattarai and Subudh, 2018
		Root biomass	Root fresh biomass	*qFRM1.36*	N-22	1	180.0	6.7	Bhattarai and Subudh, 2018
			Root dry biomass	*qDRM1.11*	Cocodrie	1	68.0	5.5	Bhattarai and Subudh, 2018
				qDRM1.37	N-22	1	184.0	7.6	Bhattarai and Subudh, 2018
	Super Basmati×IR55419-04	Root biomass	Root dry weight	*qTRDW3*	IR55419-04	3	236.3	20.9	Sabar *et al.,* 2019
		Root length	Deep root length	*qDRL3*	IR55419-04	3	226.3	29.51	Sabar *et al.,* 2019
				qDRL9	IR55419-04	9	50.0	9.11	Sabar *et al.,* 2019
				qDRL11	IR55419-04	11	56.0	3.8	Sabar *et al.,* 2019

		Root volume	Deep root volume	*qDRV3*	-	3	232.3	25.78	Sabar *et al.,* 2019
		Root area	Deep root surface area	*qDRSA3.1*	Super Basmati	3	136.7	13.62	Sabar *et al.,* 2019
				qDRSA3.2	IR55419-04	3	228.3	32.09	Sabar *et al.,* 2019
		Root diameter	Deep root diameter	*qDRD3.1*	Super Basmati	3	136.8	18.34	Sabar *et al.,* 2019
				qDRD3.2	IR55419-04	3	242.3	25.43	Sabar *et al.,* 2019
	IR64× Kinandang Patong	Deep rooting	Root angle	*Dro1*	Kinandang Patong	9	44 (approx.)	66.6	Uga *et al.,* 2011 and 2013a
wheat	W7984×Opata 85	Root length	Root length	*Qrls.uwa-1AS*	W7984	1	36.6	9	Ayalew *et al.,* 2017
				Qrls.uwa-3AL	W7984	3	92.2	13	Ayalew *et al.,* 2017
				Qrls.uwa-7BL.1	Opata 85	7	38.9	12	Ayalew *et al.,* 2017
				Qrls.uwa-7BL.2	Opata 85	7	44.7	13	Ayalew *et al.,* 2017
		Root biomass	Root dry weight	*Qrdws.uwa-4AL*	Opata 85	4	65.7	10	Ayalew *et al.,* 2017
				Qrdws.uwa-5AL	W7984	5	53.8	15	Ayalew *et al.,* 2017
	Hanxuan 10 ×Lumai 14 DH lines	Root number	Seminal root number	*QSRN.cgb-2B*	Hanxuan 10	2	0.3	8.67	Liu *et al.,* 2013
				QSRN.cgb-3B	Hanxuan 10	3	-1.7*	9.08	Liu *et al.,* 2013
		Root length	Total root length	*QTRL.cgb-3B*	Hanxuan 10	3	-7.7*	13.81	Liu *et al.,* 2013
		Root area	Projected root area	*QPRA.cgb-3B*	Hanxuan 10	3	-0.6*	5.97	Liu *et al.,* 2013
			Root surface area	*QRSA.cgb-3B*	Hanxuan 10	3	-0.6*	5.97	Liu *et al.,* 2013
		Root angle	Seminal root angle	*QSRA.cgb-2B*	Hanxuan 10	2	3.0*	10.66	Liu *et al.,* 2013
	SeriM82×Hartog	Root angle	Seminal root angle	*QRA.qgw-2A*	SeriM82	2	80.2	-	Christopher *et al.,* 2013

				QRA.qgw-3D	SeriM82	3	9.1	-	Christopher *et al.,* 2013
				QRA.qgw-6A	SeriM82	6	5.3	-	Christopher *et al.,* 2013
				QRA.qgw-6B.2	SeriM82	6	149.9	-	Christopher *et al.,* 2013
		Root number	Seminal root number	*QRN.qgw-4A.1*	SeriM82	4	3.3	-	Christopher *et al.,* 2013
				QRN.qgw-6A	SeriM82	6	40.7	-	Christopher *et al.,* 2013
	Devon×Syn084	Root volume	Root volume	*QRv.D84-2A.a*	Syn084	2	52.5	2.9	Ibrahim *et al.,* 2012
				QRv.D84-5D.a	Syn084	5	82.0	3.2	Ibrahim *et al.,* 2012
				QRv.D84-3A.a	Syn084	3	83.3	0.8	Ibrahim *et al.,* 2012
		Root area	Surface root area	*QSra.D84-2A.a*	Syn084	2	52.5	2.5	Ibrahim *et al.,* 2012
				QSra.D84-2D.a	Syn084	2	48.2	0.6	Ibrahim *et al.,* 2012
				QSra.D84-5B.a	Syn084	5	14.3	0.6	Ibrahim *et al.,* 2012
				QSra.D84-7D.a	Syn084	7	28.2	0.7	Ibrahim *et al.,* 2012
		Root length	Total root length	*QTrl.D84-7A.a*	Syn084	7	144.0	3.2	Ibrahim *et al.,* 2012
				QTrl.D84-2D.a	Syn084	2	48.2	0.7	Ibrahim *et al.,* 2012
				QTrl.D84-5B.a	Syn084	5	14.3	0.7	Ibrahim *et al.,* 2012
				QTrl.D84-7D.a	Syn084	7	28.2	0.7	Ibrahim *et al.,* 2012
	WCC	Root length	Root length	*wPt6278*	-	2	83.9	17.0	Ayalew *et al.,* 2018
				wPt1159	-	3	44.4	14.0	Ayalew *et al.,* 2018
Maize	DTP79× B73	Root density	Root density	-	DTP79	1	-	24.2	Rahman *et al.,* 2011
	DH1M × T877	Root angle	Crown root angle	*CRA2*	DH1M	1	401.0	4.2	Li *et al.,* 2018
		Root diameter	Crown root diameter	*CRD1*	-	2	289.6	2.7	Li *et al.,* 2018
		Root length	Crown root length	*CRL1*	T877	1	76.9	3.8	Li *et al.,* 2018

			Primary root length	*PRL2*	DH1M	6	22.5	0.8	Li *et al.,* 2018
				PRL3	DH1M	10	36.3	0.1	Li *et al.,* 2018
			Seminal root length	*SRL2*	T877	2	187.8	1.2	Li *et al.,* 2018
				SRL7	T877	4	275.5	3.1	Li *et al.,* 2018
				SRL9	T877	10	48.6	6.6	Li et al., 2018
Barley	Scarlett×ISR42-8	Root length	Root length	*QRl.S42.2H*	Scarlett	2	41.1	6.1	Arifuzzaman *et al.,* 2014
				QRl.S42.3H	Scarlett	3	118.72	5.5	Arifuzzaman *et al.,* 2014
				QRl.S42IL.5H.b	Scarlett	5	203.85-231.75	41.8 (RP)	Naz *et al.,* 2014
				QRl.S42IL.5H.a	Scarlett	5	105.91-109.27	41.6 (RP)	Naz *et al.,* 2014
		Root biomass	Root dry weight	*QRdw.S42.1H.a*	Scarlett	1	39.0	6.5	Arifuzzaman *et al.,* 2014
				QRdw.S42.1H.b	Scarlett	1	123.09	7.9	Arifuzzaman *et al.,* 2014
				QRdw.S42.5H	Scarlett	5	126.77	4.2	Arifuzzaman *et al.,* 2014
				QRdw.S42.7H	Scarlett	7	42.5	6.9	Arifuzzaman *et al.,* 2014
				QRdw.S42IL.1 H	Scarlett	1	24.17-40.51	78.8 (RP)	Naz *et al.,* 2014
				QRdw.S42IL.6H	Scarlett	6	133.29-133.47	66.6 (RP)	Naz *et al.,* 2014
				QRdw.S42IL.5H	Scarlett	5	203.85-231.75	63.6 (RP)	Naz *et al.,* 2014

		Root volume	Root volume	*QRv.S42IL.1H*	Scarlett	1	24.17-40.51	53.6 (RP)	Naz *et al.*, 2014
				QRv.S42IL.2H	Scarlett	2	197.39-206.17	50.7 (RP)	Naz *et al.*, 2014
	BAP	Root biomass	Root dry biomass	*QRdw.2H*	-	2	106.8	18.59	Reinert *et al.*, 2016
				QRdw.2H	-	5	95.00	24.93	Reinert *et al.*, 2016
				QTL-4H-2	-	4	17.8	-	Abdel-Ghani *et al.*, 2019
				QTL-2H-4	-	2	23.2	-	Abdel-Ghani *et al.*, 2019
		Root length	Root length	*QTL-1H-4*	-	1	66.1	-	Abdel-Ghani *et al.*, 2019
				QTL-2H-4	-	2	23.8	-	Abdel-Ghani *et al.*, 2019
				QTL-3H-8	-	3	128.6	-	Abdel-Ghani *et al.*, 2019
		Root angle	Tiller nodal root growth angle	*QTL-3H_2*	-	3	52.03	3.8	Oyiga *et al.*, 2019
		Root number	Nodal root number at the tiller	*QTL-7H*	-	7	34.00	3.5	Oyiga *et al.*, 2019
		Xylem vessel size	Tiller nodal root xylem vessel area	*QTL-5H_2*	-	5	28.26	3.2	Oyiga *et al.*, 2019
				QTL-3H_3	-	3	58.0	2.1	Oyiga *et al.*, 2019
Sorghum	B923296 × SC170-6-8	Root angle	Nodal root angle	*qRA1_5*	B923296	5	51.8	10.01	Mace *et al.*, 2012
				qRA2_5	SC170-6-8	5	34.0	29.78	Mace *et al.*, 2012
				qRA1_8	SC170-6-8	8	25.4	6.72	Mace *et al.*, 2012
				qRA1_10	SC170-6-8	10	208.4	11.65	Mace *et al.*, 2012

		Root biomass	Root dry weight	*qRDW1_2*	-	2	216.8	13.05	Mace *et al.,* 2012
				qRDW1_5	-	5	101.7	12.06	Mace *et al.,* 2012
				qRDW1_8	-	8	123.3	6.97	Mace *et al.,* 2012

Chr. No.; Chromosome number; PVE, phenotypic variation explained the QTL; RP, Relative trait performance of the S42IL compared to Scarlett; *, Genetic distance between putative QTL peak value and the nearest flanking marker. Positive values are between the QTL and the left flanking marker, negative values are between the QTL and the right flanking marker; BPM, Bi-parental mapping; GWAS, Genome-wide association studies; WCC, wheat core collection; BAP, Barley association panel.

References:

Abdel-Ghani AH, Sharma R, Wabila C, Dhanagond S, Owais SJ, Duwayri MA, et al. 2019. Genome-wide association mapping in a diverse spring barley collection reveals the presence of QTL hotspots and candidate genes for root and shoot architecture traits at seedling stage. BMC Plant Biology **19**, 216.

Arifuzzaman M, Sayed MA, Muzammil S, Pillen K, Schumann H, Naz AA, Léon J. 2014. Detection and validation of novel QTL for shoot and root traits in barley (*Hordeum vulgare* L.). Molecular Breeding **34 (3),** 1373-1387.

Ayalew H, Liu H, Börner A, Kobiljski B, Liu C, Yan G. 2018. Genome-Wide association mapping of major root length QTLs under peg induced water stress in wheat. Frontiers in Plant Science **9**, 1759.

Ayalew H, Liu H, Yan G. 2017. Identification and validation of root length QTLs for water stress resistance in hexaploid wheat (*Titicum aestivum* L.). Euphytica **213,**126.

Bhattarai U, Subudhi PK. 2018. Identification of drought responsive QTLs during vegetative growth stage of rice using a saturated GBS-based SNP linkage map. Euphytica **214,** 38.

Catolos M, Sandhu N, Dixit S, Shamsudin N, Naredo M, McNally KL, Henry A, Diaz MG, Kumar A. 2017. Genetic loci governing grain yield and root development under variable rice cultivation conditions. Frontiers in Plant Science **8,** 1763.

Christopher J, Christopher M, Jennings R, Jones S, Fletcher S, Borrell A, Manschadi AM, Jordan D, Mace E, Hammer G. 2013. QTL for root angle and number in a population developed from bread wheat (*Triticum aestivum*) with contrasting adaptation to water-limited environments. Theoretical and Applied Genetics **126,** 1563–1574.

Han J, Shin N, Jang S, Yu Y, Chin JH, Yoo S. 2018. Identification of quantitative trait loci for vigorous root development under water-deficiency conditions in rice. Plant Breeding and Biotechnology **6(2),** 147–158.

Ibrahim SE, Schubert A, Pillen K, Léon, J. 2012. QTL analysis of drought tolerance for seedling root morphological traits in an advanced backcross population of spring wheat. International Journal of Agricultural Science **2(7),** 619–629.

Li P, Zhang Y, Yin S, Zhu P, Pan T, Xu Y, Wang J, Hao D, Fang H, Xu C, Yang Z. 2018. QTL-by-environment interaction in the response of maize root and shoot traits to different water regimes. Frontiers in Plant Science **9,** 229.

Liu X, Li R, Chang X, Jing R. 2013. Mapping QTLs for seedling root traits in a doubled haploid wheat population under different water regimes. Euphytica **189(1),** 51–66.

Mace ES, Singh V, Van Oosterom EJ, Hammer GL, Hunt CH, Jordon DR. 2012. QTL for nodal root angle in sorghum (*Sorghum bicolor* L. Moench) co-locate with QTL for traits associated with drought adaptation. Theoretical and Applied Genetics **124,** 97-109.

Naz AA, Arifuzzaman M, Muzammil S, Pillen K, Léon J. 2014. Wild barley introgression lines revealed novel QTL alleles for root and related shoot traits in the cultivated barley (*Hordeum vulgare* L.). BMC Genetics **15,** 107.

Oyiga BC, Palczak J, Wojciechowski T, Lynnch JP, Naz AA, Leon J, Ballvora A. 2019. Genetic components of root architecture and anatomy adjustments to water-deficit stress in spring barley. Plant, Cell & Environment **43(3),** 1–20.

Rahman H, Pekic S, Lazic-Jancic V, Quarrie SA, Shah SMA, Pervez A, Shah MM. 2011. Molecular mapping of quantitative trait loci for drought tolerance in maize plants. Genetics and Molecular Research **10 (2),** 889-901.

Reinert S, Kortz A, Léon J, Naz AA. 2016. Genome-wide association mapping in the global diversity set reveals new QTL controlling root system and related shoot variation in barley. Frontiers in Plant Science **7,** 1061.

Sabar M, Shabir G, Shah SM, Aslam K, Naveed SA, Arif M. 2019. Identification and mapping of QTLs associated with drought tolerance traits in rice by a cross between Super Basmati and IR55419-04. Breeding Science **69(1),** 169–178.

Uga Y, Okuno K, Yano M. 2011. *Dro1*, a major QTL involved in deep rooting of rice under upland field conditions. Journal of Experimental Botany **62,** 2485–2494.

Uga Y, Sugimoto K, Ogawa S, Rane J, Ishitani M, Hara N, Kitomi Y, Inukai Y, Ono K, Kanno N. et al. 2013a. Control of root system architecture by *DEEPER ROOTING 1* increases rice yield under drought conditions. Nature Genetics **45,** 1097–1102.
